# THE INTERSECTION OF LIVING ALONE, DEPRESSION, AND RESIDENTIAL ENVIRONMENT AMONG OLDER KOREANS WITH DISABILITIES

**DOI:** 10.1093/geroni/igae098.2685

**Published:** 2024-12-31

**Authors:** Jihyeong Jeong

**Affiliations:** University of Maryland Baltimore, Baltimore, Maryland, United States

## Abstract

The ecological theory of aging explains how the interactions between individuals’ capabilities and their environment affect psychological well-being. The risk of depression rises among older adults living alone, and this vulnerability is further accentuated for those with disabilities, as their residential environments may not adequately compensate for the multifaceted challenges associated with impairments. The current study analyzed a sample comprised of 2,220 older adults with disabilities, aged 60 years or over, selected from the first wave of the 2021 Disability and Life Dynamics Panel of South Korea. As a cross-sectional study, a linear regression was performed controlling for gender, job status, disability type, and disability level. The findings reported that (a) as the perceived housing environment goes down by 1 unit, depression goes up by.20 (p <.001) and (b) older adults who live alone were associated with higher levels of depression compared to those who do not live alone (Beta = 1.36; p <.001). Aligning with the ecological theory of aging, the results underscore the significance of the residential environment in the psychological well-being of older adults. Furthermore, the positive correlation between living alone and higher levels of depression implies the importance of social support systems in mitigating depressive symptoms. Given these results, interventions aimed at improving the living conditions and social support networks of older adults who live alone with disabilities stand to produce meaningful improvements in their mental health.

